# Superficial siderosis in cerebral amyloid angiopathy

**DOI:** 10.4103/0972-2327.78056

**Published:** 2011

**Authors:** Nisar Ahmad Wani, Tasleem L. Kosar, Aijaz A. Rawa, Abdul K. Qayum

**Affiliations:** Department of Radiodiagnosis and Imaging, Sher-I- Kashmir Institute of Medical Sciences, Srinagar, Jammu & Kashmir, India; 1 Department of Neurosurgery, Sher-I- Kashmir Institute of Medical Sciences, Srinagar, Jammu & Kashmir, India

## Introduction

**Figure 1:**
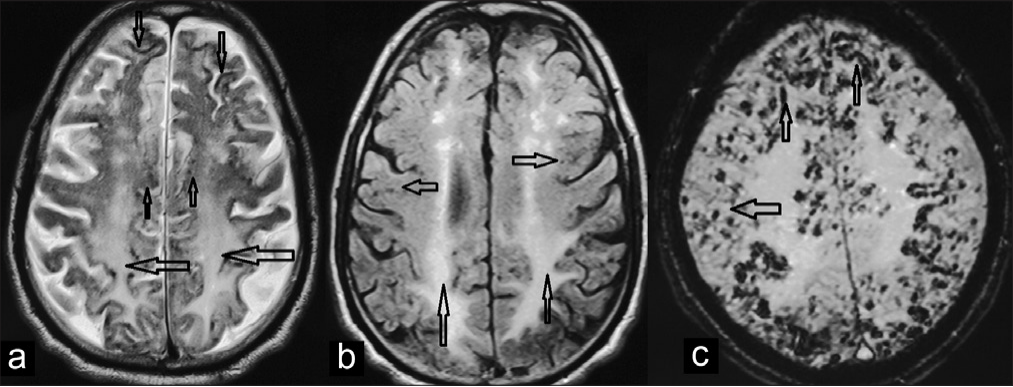
(a) T2 weighted axial MR image showing gyriform hypointense signal intensity foci along the surface of frontal and parietal cerebral convexities of both cerebral hemispheres (downward arrows). Small rounded signal void dots are seen in the cortices (upward arrows) with diffuse hyperintense signal intensity in the white matter (leftward arrows), (b) Axial T2 W fluid attenuated inversion recovery (FLAIR) image through the supraventricular level shows diffuse hyperintense signal intensity in the centrum semiovale bilaterally (upward arrows); small subcortical signal void foci are also seen (right and leftward arrows), (c) Susceptibility weighted (SWI) axial MR image through cerebral convexities above the ventricular level showing prominent gyriform (upward arrows) and innumerable small rounded signal voids (leftward arrow) in bilateral cerebral hemispheres.

A 70-year-old non-alcoholic, non-diabetic, and non-smoker right hand dominant man presented with a 5 year history of dementia with progressive aphasia. On examination, blood pressure was observed to be 140/80 mm Hg; neurological examination was normal whereas cognitively he had impairment of memory and language in the form of non-confluent aphasia. The erythrocyte sedimentation rate, C-reactive protein, lupus anticoagulant, and anticardiolipin antibodies; and prothrombin and partial thromboplastin times were all within normal limits. Magnetic resonance imaging (MRI) examination of brain was performed with a 1.5 tesla MR imager using T1 weighted (T1 W) and T2 W sequences in various planes; T2 W fluid attenuation inversion recovery (T2 W FLAIR) and susceptibility weighted imaging (SWI) sequences were also performed. MR imaging with conventional T1 W and T2 W sequences revealed generalised atrophy of brain. T2 W and FLAIR images showed diffuse and symmetric confluent hyperintense signal intensity in the periventricular white matter of both cerebral hemispheres [Figures 1]. T2 W axial MR images showed innumerable small (up to 1 cm in diameter) subcortical signal void foci in the bilateral cerebral hemispheres. Thin gyriform hypointensities were seen on T2W images in the cerebral convexities of frontal and parietal regions of both hemispheres [Figure 1]. Small rounded subcortical and gyriform convexity hypointense signal intensities on T2W images became more prominent and extensive on SWI suggesting these to be hemosiderin deposits. Basal ganglia, thalami, and internal capsule were all normal. Clinical features and MRI findings were compatible with a diagnosis of probable cerebral amyloid angiopathy (CAA) with leukoencephalopathy and superficial siderosis.

## Discussion

Superficial siderosis is a radiologic or pathologic demonstration of hemosiderin in the subpial layers of brain and spinal cord.[1] It is thought to result from chronic or recurrent bleeding into the subarachnoid space due to trauma, brain tumors, vascular malformations of central nervous system, and intracranial aneurysms. Patients present with classical triad of progressive sensorineural hearing loss, cerebellar ataxia, and pyramidal signs.[1] With the advent of gradient recall echo (GRE) MRI, superficial siderosis is being recognised in asymptomatic elderly persons and in patients with Alzheimer disease (AD).[2] More recently a link has been suggested between superficial siderosis and CAA.[3]

CAA is characterised by deposition of amyloid in the walls of small blood vessels and capillaries of leptomeninges and cortex of brain.[4,5] It is an age related condition with increasing prevalence in elderly, which is present in almost all patients with AD. Usual presentation is with stroke due to intracerebral bleed or cognitive impairment when it is associated with white matter signal alteration on MRI consequent to chronic ischemia.[6] Intracerebral hemorrhages in CAA are typically superficial in cortical and subcortical distribution sparing deep gray matter.[5] Bleeding results from weakening of the vessel walls due to deposited amyloid protein in media. Superficial location may allow seepage of blood into the subarachnoid space of the brain convexity, alternately blood may leak from subarachnoid space arteries or arterioles.[3,6] Impaired clearance of the hemorrhage due to genetic predisposition may contribute to superficial deposition of hemosiderin along the subpial layer of brain.[3,7]

Radiological demonstration of multiple primarily cortical and corticosubcortical (lobar) hemorrhages in the brain of elderly patients is pointer towards a diagnosis of CAA.[8] According to Boston criteria a probable diagnosis of CAA is made in elderly patients with at least two acute or chronic lobar hemorrhagic lesions without any other definite cause of intracerebral hemorrhage like prior trauma, ischemic stroke, CNS tumor, vascular malformation, or bleeding diathesis.[9] MRI using GRE sequence has increased sensitivity for detecting intracerebral bleed based on the susceptibility effect caused by hemosiderin. Paramagnetic effect by hemosiderin in microhemorrhages causes variation in local magnetic field and local reduction of T2*, which results in signal loss on GRE images. SWI is a three dimensional, velocity-compensated, GRE sequence that combines both magnitude information with phase information to accentuate the visibility of susceptible foci maximising the detection of lobar hemorrhages in CAA.[10] GRE sequence also enhances detection of superficial siderosis, which may be located close to cortical hemorrhages in more advanced CAA. Striking gyriform appearance of superficial siderosis with numerous adjacent corticosubcortical signal voids due to microhemorrhages were more prominent on SWI as compared to conventional T2W images in our case. This identification of superficial siderosis, enhanced by GRE imaging, may further facilitate the non-invasive imaging diagnosis of CAA with MRI.[3,7] Other MRI findings in CAA include cerebral atrophy and leukoencephalopathy.[8]

Identification of CAA with evidence of extensive lobar microhemorrhages and superficial siderosis on MRI as in our case, make it extremely necessary to decide about the judicious use of anticoagulation and antiplatelet therapy for preventing ischemic events prevalent in this age group. Management of CAA is currently centred on preventing further bleeding episodes and disease progression. MRI can be used for monitoring the progress.[8]

We conclude that presence of superficial siderosis may be related to severity of CAA. Recognition of superficial siderosis on MRI may provide further support to imaging diagnosis of CAA, which is enhanced by using SWI.

## References

[1] Kumar N (2010). Neuroimaging in Superficial Siderosis: An In-Depth look. AJNR Am J Neuroradiol.

[2] Vernooij MW, Ikram MA, Hofman A, Krestin GP, Breteler MM, van der Lugt A (2009). Superficial siderosis in the general population. Neurology.

[3] Feldman HH, Maia LF, Mackenzie IR, Forster BB, Martzke J, Woolfenden A (2008). Superficial siderosis: A potential diagnostic marker of cerebral amyloid angiopathy in Alzheimer disease. Stroke.

[4] Greenberg SM (1998). Cerebral amyloid angiopathy: Prospects for clinical diagnosis and treatment. Neurology.

[5] Lee SH, Kim SM, Kim N, Yoon BW, Roh JK (2007). Cortico-subcortical distribution of microbleeds is different between hypertension and cerebral amyloid angiopathy. J Neurol Sci.

[6] Thal DR, Griffin WS, de Vos RA, Ghebremedhin E (2008). Cerebral amyloid angiopathy and its relationship to Alzheimer’s disease. Acta Neuropathol.

[7] Linn J, Herms J, Dichgans MJ, Brückmann H, Fesl G, Freilinger T (2008). Subarachnoid hemosiderosis and superficial cortical hemosiderosis in cerebral amyloid angiopathy. AJNR Am J Neuroradiol.

[8] Chao CP, Kotsenas AL, Broderick DF (2006). Cerebral Amyloid Angiopathy: CT and MR Imaging Findings. Radiographics.

[9] Knudsen KA, Rosand J, Karluk D, Greenberg SM (2001). Clinical diagnosis of cerebral amyloid angiopathy: Validation of the Boston criteria. Neurology.

[10] Haacke EM, delProposto ZS, Chaturvedi S, Sehgal V, Tenzer M, Neelavalli J (2007). Imaging cerebral amyloid angiopathy with susceptibility-weighted imaging. AJNR Am J Neuroradiol.

